# New Perspectives on the Organization of Living Tissue and the Ongoing Connective Tissue/Fascia Nomenclature Debate, as Revealed by Intra-Tissue Endoscopy That Provides Real-Time Images During Surgical Procedures

**DOI:** 10.3390/life15050791

**Published:** 2025-05-15

**Authors:** Jean Claude Guimberteau, Elias T. Sawaya, Colin Armstrong

**Affiliations:** 1 Surgeon Aquitaine Hand and Upper Extremity Institute, 56 Allée des Tulipes, 33600 Pessac, France; esawaya.md@gmail.com; 2 Osteopathic Centre, 1732 Voie Aurelienne, 13450 Grans, France; arm.colin@gmail.com

**Keywords:** fascia, connective tissue, fibrillar network, microvolumes, cell microenvironment, force absorption system, intra-tissue endoscopy

## Abstract

Intra-tissue endoscopy, providing real-time images at all scales, from macroscopic to microscopic, from inside living tissue during surgical procedures, has revealed the existence of a body-wide fibrillar architecture that extends from the surface of the skin to the cell. Different types of cells are housed within this fibrillar architecture and gather together to carry out specific functions. This challenges the commonly accepted notion of the organization of living matter that associates separate organs with connective tissue packaging. We are thus confronted with the global nature of the living human body and its vital processes. This paper sets out to describe the architecture of this fibrillar network which could be assimilated with the fascial tissue and which attributes a more constitutive role to connective tissue. It also demonstrates how movements within this fibrillar network can occur with minimal local distortion while maintaining tissue continuity. The authors propose that the gliding of tissues can be explained by the existence of a highly adaptable fibrillar network that enables the gliding of distinct anatomical structures such as tendons and muscles, without any dynamic influence on the surrounding tissues. The authors propose a new model of tissue movement based on the observation of a ubiquitous dynamic polyhedric fibrillar network with an apparently dispersed and complex pattern of organization, that forms fluid-filled microvolumes, and is found everywhere in the human body. Furthermore, this fibrillar network appears to act as a force absorption system, in addition to providing a framework or scaffolding for cells throughout the body. Observation during intra-tissue endoscopy suggests that this fundamental architectural organization extends into the extracellular matrix that is the natural environment of all cells in the living body, regardless of their size, location or specific function.

## 1. Introduction

Historically, the vast majority of anatomical research has been performed on cadavers, or by studying tissue samples under the microscope. This means that the study of living matter has been carried out on dead and inert structures. The authors of this paper consider this to be a paradox. Recent fascia research, as interesting and relevant as it may be, is still being carried out on dead tissue samples under a microscope, by ultrasound scans or other types of imaging that provide only two-dimensional images, and give no information of the three-dimensional architecture or mobility of living tissue, in situ, inside the human body [1,2,3].

Many structures glide under the surface of the skin and yet maintain continuity with surrounding tissues. This gliding motion is largely attributed to “loose areolar tissue”, or connective tissue. Currently, these structures are poorly defined, and the understanding of how these tissues move is unclear. Anatomical texts characterize all the intermediate tissues as “fascia” or connective tissue. This term encompasses a wide range of tissue organizations and leads to confusion and misinterpretation because they are definitions derived essentially from the dissection of cadavers.

On the other hand, dense fascial structures are generally accepted to be specialized in force transmission [4]. They are composed predominantly of type I collagen fibers. The organization of these fibers is visible to the naked eye as parallel bundles.

These different types of fascia, known also as connective tissue, are considered to be filling or packaging tissues, providing physical links between the so-called “noble” organs, but their mechanical behavior has never been studied by human intra-tissue endoscopy in vivo, and in situ inside the living body [5,6,7,8,9,10].

The use of intra-tissue endoscopes with miniaturized high-magnification cameras during surgical procedures has introduced a new method of observation of living tissue. To the best of our knowledge, this innovative exploration of human living tissue is unique. This original research offers a new perspective on the organization of living matter.

This paper is based on the in vivo microscopic examination of a fibrillar network that is observable during surgical procedures using intra-tissue endoscopy, with or without the application of a tourniquet.

In light of these observations, the authors propose the existence of a continuous body-wide multifibrillar network. Three-dimensional modeling of this network has been produced in an attempt to illustrate the behavior and organization of this body-wide multifibrillar network, and the role it plays in movement. The insights gained from this research appear to confirm the concept of the body as a global dynamic functional fibrillar scaffolding.

We propose a complete change of paradigm. The implications of such a paradigm shift would be multiple, profound and wide-ranging, especially in the fields of anatomy, surgery and manual therapy.

The initial aim of this research was primarily surgical. Indeed, given the poor functional results obtained in tendon reconstructions using a two-stage surgical technique, creating recovery times and work stoppages of between 6 and 12 months, it was decided to develop a technique that could be carried out in one stage.

To do this, it was necessary to use well-vascularized tendon transfers and not simple non-vascularized grafts that created adhesions and gave very poor results. For this, too, it was necessary to better understand tendon vascularization and the tendon gliding system, particularly the finger flexors, but also the tendon-muscle junction and the finger flexor muscles between them [11,12,13,14,15].

This was the initial objective.

But given the surprising variety of observations and their notable differences with the teaching received and given, it was decided to expand the field of investigation to all the anatomical areas where the microsurgical transplant surgery was applicable. The priority remained surgical, however, because the well-being of the patient is central to this study. This exploratory research was therefore carried out according to the requirements of the specific surgical indications of each surgical intervention. It could not have been otherwise. This explains the impossibility of presenting a well-programmed study, with a planned methodology, as it is possible to carry out in the laboratory with cloned mice or rats. However, this study of intra-tissue endoscopy in humans on 1305 patients is unique for the moment, and the observations and interpretations cannot be compared with any other previous research or studies.

Three simple observations are often taken for granted, but are poorly explained by simplistic mechanical models:

When you see all these components under the skin together and you think of the complex movements of a pianist’s fingers, all the fingers gliding with different pressures, without getting in each other’s way, delicately and then immediately moving on to another combination of movements. Or, when the flexor tendons glide within the palm of the hand, they move up to 3 cm, and there is no evidence of this motion on the surface of the hand, so there must be absorption of the force.

**Figure 1:**
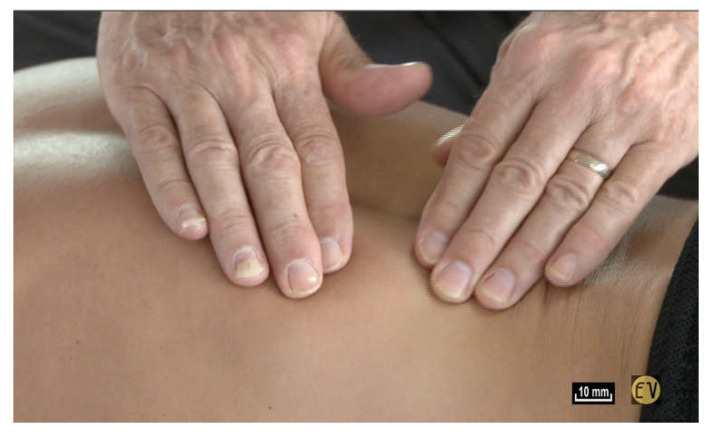
(Video S1) After massage, the skin always returns to the same place without any modification—there is a real tissue memory.

Or, when you impose simple traction by lifting the skin, and you let go, the skin always returns to the same place without any modification; there is a real tissue memory that is efficient and always enables the underlying structures to return to their original configuration (Figure 1).

How is this achieved without rupture or failure?

Traditional teaching proposes a few simple explanations using mechanistic concepts involving layers, sub-layers, stratifications and virtual spaces, but which are never really observed inside the living body.

The generic term “the connective tissue” implies that it connects anatomical structures and acts as a filling or packaging binding material. But what is connective tissue?

## 2. Materials and Methods

**Figure 2:**
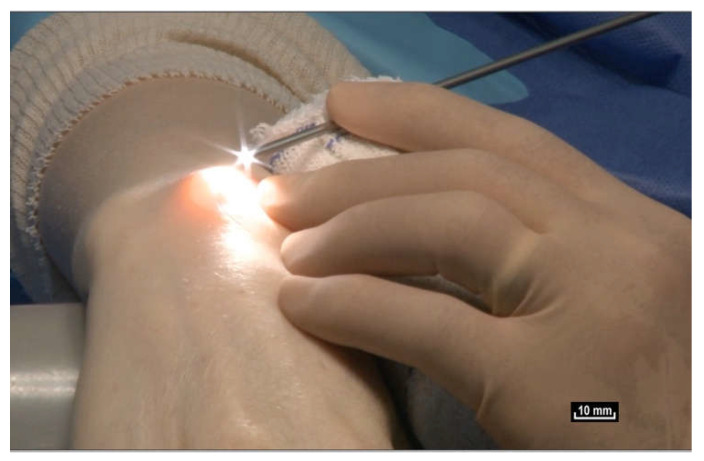
(Video S2) Use of the endoscope during surgery. Viewing the video in real time on a screen in the operating theatre during surgical endoscopy.

To find out, we had to review how the gliding mechanism works by using an innovative new technology, intra-tissue endoscopy. This involves placing the endoscope *inside* the tissues under observation during surgery. Intra-tissue endoscopy at this level of observation was made possible by the use of an endoscopic contact probe (ref. Storz, Hamou, 26120BHA Hopkins system, Karl Storz, Tuttlingen, Germany, 30° angle view, 2.9 mm diameter, 30 cm length and a magnifying capacity of 60×), connected to a high-definition processor (ref. Karl Storz “Image One” camera, Karl Storz, Tuttlingen, Germany) coupled to a cold light source (ref. Xenon Nova 201215, Karl Storz, Tuttlingen, Germany) (Figure 2).

The sequences were recorded with a video recorder (ref. KIPRO AJA HDV, AJA, Grass Valley, CA, USA, 10 codecs Linear PCM, Timecode, Apple ProRes 422, Apple Inc., Cupertino, CA, USA). With this new technology, it is possible to obtain levels of detail never achieved before. The time set aside for filming was limited to 10 min so that the work of the surgical team was not disturbed. These surgical interventions were performed either with or without a surgical tourniquet. The use of a tourniquet provides a bloodless field, which allows detailed observation. Many factors can disturb the filming process. The main factor of disturbance is bleeding. The use of a tourniquet for the limbs is the solution, but the pressure must average about 18 mmHg. Maximum stability is required to avoid jerking of the endoscopic lens. Some tissues are more opaque than others, and while some provide the surgeon with superb images, others are totally unusable, for which there is no satisfactory explanation. It is necessary to clean the optics regularly because fragments of lipid particles, fog or droplets appear in certain patients very quickly. Finally, focusing with a wheel is very delicate because the depth of the field is very short, and the images become blurred very easily.

### 2.1. Patient Selection

This was based on the length of the surgery. Only patients whose operation protocol would last at least 30 min were selected. Another selection criterion was the size of the surgical incision. It is difficult to perform intra-tissue endoscopy through an incision of less than 3 cm. All filming was carried out during or after planned surgical interventions, with the consent of each patient. The time set aside for filming was limited to 10 min so that the work of the surgical team was not disturbed.

Three types of surgical procedures were selected for this research project.

(1) Superficial incision with a tourniquet at the proximal end of the limb. The initial incision was made through the skin to the hypodermis. Then, the area immediately below the hypodermis was exposed using small skin hooks that allowed the fibrillar network to be revealed. No surgical detachment was made beforehand in order to ensure that the tissue remained intact. The tip of the endoscope was then placed directly above this area, and the recording started. No filming scenario was prepared beforehand. After about 10 min of filming, the patient’s surgical procedure was carried out.
(2) Deep incision with a tourniquet at the proximal end of the limb. This was used during longer surgical procedures, and exposed deeper structures such as muscles, nerves and bones. The same procedure as above was adopted. That is, filming was carried out for about 10 min before surgery.
(3) Deep incision without a tourniquet. In these cases, it is essential to perform excellent hemostasis with a bipolar scalpel to avoid bleeding and prevent the tissues from being infiltrated with hemoglobin.

The images were analyzed the same evening and classified by date, the nature of the tissues and any remarkable events or observations.

This methodology requires no exceptional surgical skills and can be reproduced by any surgeon.

### 2.2. Statistics

A total of 1305 patients were included in this research project, with written consent, over a period of 15 years.

Intra-tissue endoscopy was performed on an average of 90 patients a year during this period. This represents a total of about 120 h of video (an average of 40 days of filming per year.

Sex: 65% female patients; 35% male patients.

Age: 10 to 82 years old.

Localizations: Forearm 28% • Elbow18% • Abdomen• 15% • Hand 19% • Thorax 13% • Lumbar and sacral area 4% • Lower limb 3% • Scalp 1%.

A total of 1500 video sequences and 200 explanatory animations are available for scientific research on the website www.endovivo.com (accessed on 1 March 2021).

**Figure 3:**
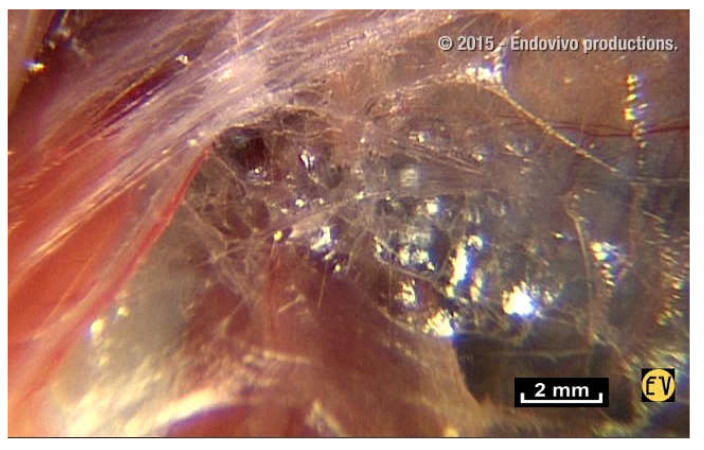
(Video S3) There are no empty spaces in the body. All available space is occupied. A world of fibers. Fibers are everywhere, in every nook and cranny. There is no apparent order. ×20.

The result was the discovery of the mostly unknown, uncharted and unexplored aspect of living connective tissue/fascia. Vibrant colors, such as blues, reds, browns and yellows, with surfaces reflecting the light of the endoscope, and fibers and fibrils in irregular, dispersed patterns. But most importantly, this research opens up a new field in the study of anatomy: “in vivo” microanatomy (Figure 3).

### 2.3. In Vitro Study of Tissue Samples

Biopsies were taken during endoscopy sessions under visual control. The fragments were placed in a bottle of physiological serum and sent to the laboratory.

(i) Structural organization

The study was carried out by Professor J.P. Delage, senior lecturer in biology (Université Victor Segalen Bordeaux 2). Human and animal samples were compared. For example, 11 samples from the Carpi Radialis Flexor in cattle were compared with samples of the human Flexor Profundus, which is very similar. The preparation was treated with potassium bichromate, placed in formalin, and finally in caustic soda, thus allowing for a softer and more complete hydrolysis. The preparation was then frozen and freeze-dried under standard conditions for dehydration. Afterward, it was dissected under a binocular magnifying glass, and samples were taken, given a gold-metallic finish and then observed under an electron microscope [16].

(ii) Analysis of chemical components

Analysis of the chemical components of these connective tissue samples was performed by the “Institut de Biologie et de Chimie des Protéines” (IBCP) in the INSERM Laboratories (Pr Herbage, Lyon, France https://www.univ-lyon1.fr, accessed on 18 October 2004) at the Université Claude Bernard Lyon 1.

This study revealed that the sides of the intertwined microvolumes are composed of collagen fibers. These fibers are composed of collagen, mostly type 1 (23%), 3 and 4, but also elastin (4%). Their diameter ranges from a few to several dozen microns, and they vary in length, thus giving an overall disorganized, heterogeneous and chaotic appearance. The microvolumes were found to contain a highly hydrated proteoglycan gel (70%). Their lipid content (4%) is high. As a result of their strong negative charge, glycosaminoglycans attract counter-ions and water molecules into the tissue. These analyses were repeated several times, but the results varied greatly depending on the location of the biopsies, sometimes in areas close to each other, such as the elbow and wrist. Their constitution is therefore unpredictable in terms of precision. However, a constant finding was the presence of collagen 1 and proteoglycans, with no significant variation in their concentration [16].

## 3. Results and Observations

It is suggested that simply observing fascia/connective tissue in living organisms at different magnifications will provide us with knowledge that will challenge and modify our conception and understanding of living matter.

The study of tissues in cadavers, or under the microscope in a laboratory, is so different from the “in vivo” observation of living tissue “in situ”, inside living bodies, that, in our opinion, it can no longer be considered the only accepted approach to the study of human anatomy.

### 3.1. Fascia/Connective Tissue Fibers Are Arranged in a Totally Irregular, Dispersed Pattern

Widely accepted current models of force transmission in the body describe linear pathways [4].

However, when living connective tissue is studied through an endoscope at different levels of magnification, the received wisdom of an orderly arrangement of different tissue types appears to be untenable. A constant finding is a disorderly arrangement of mobile fibers of different dimensions, arranged in fractal patterns. These observations are far removed from the schematic caricatures found in many medical books.

An important observation made during intra-tissue endoscopy is that the basic fibrillar framework of this architecture never varies, whatever the type of tissue, its function or its topographical location, but with local adaptive changes.

It must be stated here that this was not a standard research project with everything planned in advance. It could be described as “investigative research” of living tissue using intra-tissue endoscopy during planned surgical interventions.

This study, therefore, started with the exploration of the skin, epidermis and dermis. However, this area is difficult to access using intra-tissue endoscopy, which does not allow for optimal observation of the mobility of the fibers present in these areas.

However, in the hypodermis, it is possible to perfectly identify the fibrillar network. Sometimes, it is so dense that it can be anatomically identified as fascia superficialis. Here, fibers can be observed in abundance between the fatty lobules. Mobile fibers of different dimensions are arranged in a disorderly, chaotic manner.

The term “chaos” is used here in reference to chaos theory in physics, which refers to systems that are apparently completely disorganized, but in which there is an underlying order within the apparent disorder [17,18,19].

These fibers are already known to be part of the connective tissue, which forms the link between the constitutive organs of the body. This observation is therefore in accordance with academic teaching [20,21,22,23,24].

This tissue, the connective tissue, is also called «packaging tissue» or sub synovial connective tissue (SSCT). It is also known as “loose fascia”, described by Still, the founder of Osteopathy [25]. Another important observation is that areas of “virtual space” between anatomical structures cannot be distinguished, as described in anatomy textbooks. Everything in the video sequences from this study is connected. There are no observable empty spaces within the living tissue observed during this study.

**Figure 4:**
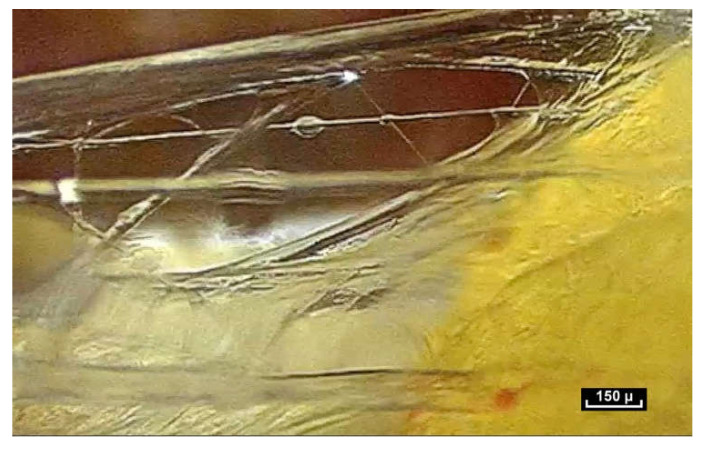
(Video S4) (×70 magnification) Fibers penetrate the fatty lobule and are dispersed between the cells. They influence the arrangement of the adipocytes inside the lobules and determine their shapes.

When the endoscope is moved closer and the magnification increases to between 25× and 60×, a major observation is that the fibers do not stop at the periphery of the fatty lobule to simply perform the role of connecting or joining structures. They can be seen to penetrate inside the lobule, and appear to play a role in determining its shape, while also providing connections with surrounding lobules. In light of these observations, it is proposed that the role of the connective tissue is much more than that of simply packaging, filling, or connecting (Figure 4).

Fibers penetrate the fatty lobule and are dispersed between the cells. There is an observable physical and mechanical link between these fibers and the cells.

### 3.2. Fascia/Connective Tissue Fibers Provide Physical Continuity—They Form a Fibrillar Continuum

The fibrillar network, which provides the architectural framework of the hypodermis, extends towards the aponeurosis, which is also called the Fascia Profunda but it is also known as “deep fascia” or “dense connective tissue”. Between the two there is an area that is almost free of cells.

It is a perfectly visible, easily observable zone made up of intercrossing fibers, surfaces that reflect the light emitted by the endoscope, and vessels. This zone is highly mobile. It is here, in the area of the hypodermis that the fibers first become visible.

On closer inspection, the distribution and arrangement of these fibers are again apparently disordered and irregular. However, this area between the hypodermis and the “deep fascia” appears to confer mobility to the skin that would enable the skin to return to its original configuration once a constraint, such as pinching and lifting the skin, is removed. An important observation here is the association of an apparently chaotic fibrillar arrangement with maximum efficiency. This can be deduced because the tissue memory is perfect. It is therefore possible to formulate the hypothesis that this is an organized fibrillar network.

**Figure 5:**
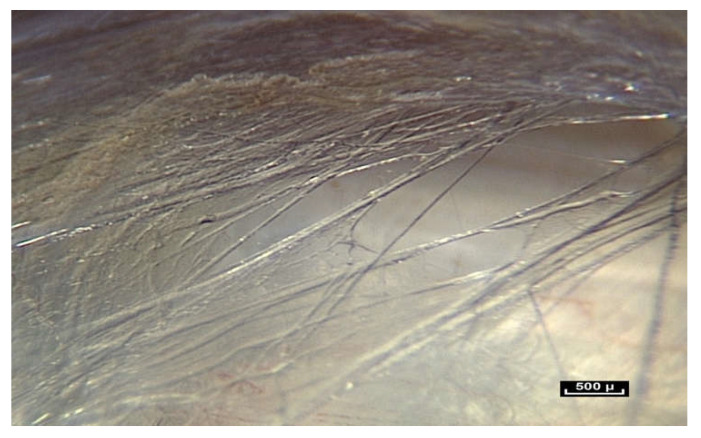
(Video S5) (×10 magnification) Muscular aponeurosis are simply densified areas of the same fibrillar network. However, their structure is in an irregular pattern and is different because of their different functional roles.

Beneath the “deep fascia” the observable fibrillar continuity extends to the epimysium, which is in continuity with the muscle. The epimysium is also made up of fibers which penetrate into the muscle. There is no visible fibrillar discontinuity, and this area is highly mobile. The area beneath the “deep fascia” appears to be composed of fibers arranged in an irregular manner, and there is no observable discontinuity of the fibers in the fibrillar network from one area to another. The “deep fascia” itself is made of collagen fibers. Its weave is also irregular, an apparently random weave obeying no obvious rules, and yet effective. Here again, the association of an apparent disorder and efficiency can be observed (Figure 5).

**Figure 6:**
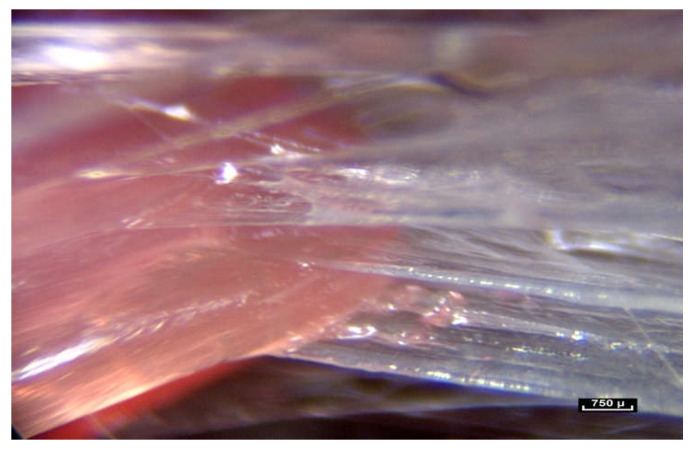
(Video S6) (×20 magnification) Perimysial fibers connecting muscle fascicles. The network of fibrils surrounds and penetrates the muscle.

When the endoscope approaches and then comes into contact with the periphery of the muscle, there is no noticeable or observable change in the continuous network of fibers, and yet here it is given a separate name—the perimysium. In reality, epi- or peri- and, endomysium are all part of the same fibrillar network, although not necessarily at the same scale of observation. However, a constant observation is the same irregular disposition of the fibers, in a dispersed pattern, in total continuity. Further magnification reveals the fascicles of the muscle. It is also possible to look closely at the perimysium. Once again it is surprising to see that the fibers of the connective tissue here, called the perimysium, not only join the fascicles together but penetrate them completely, continuing into the muscle fascicles exactly as was observed with the fatty lobules in the hypodermis (Figure 6).

This then gives rise to the same question that was formulated earlier: is connective tissue only connective?

The exploration then continues towards an area known as the endomysium, which is in fact the continuation of the perimysium, itself connective tissue, but on a smaller scale, around 10 microns. Here, the use of the contact endoscope is more difficult because the structures are fragile. Our research using an electron microscope has confirmed the above observations [16]. The endomysial fibers come into contact with the sarcomeres very often and appear to extend into the cell. Once the muscle cell has been removed by a chemical process, the endomysial envelope is comparable to what has been observed using intra-tissue endoscopy at the intermediate, mesoscopic level—a network of fibrils in apparent disorder, forming a polyhedric irregular framework.

But before describing the observations at high magnification, two important observations must be highlighted.

### 3.3. Fascia/Connective Tissue Fibers Are Subject to Permanent Endogenous Tension

**Figure 7:**
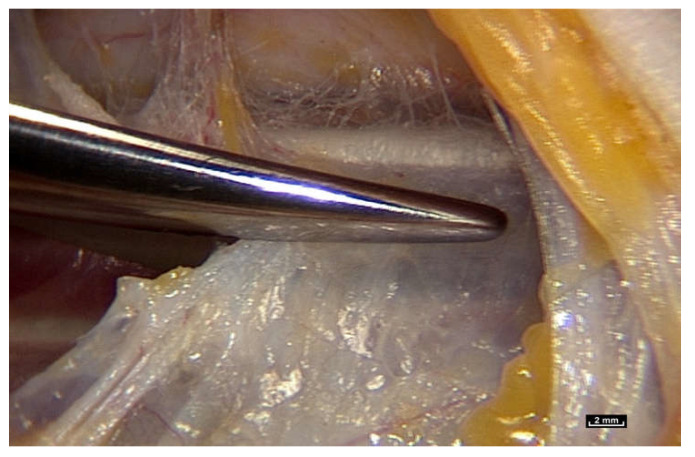
(Video S7) During section of an aponeurosis, the edges retract like an elastic band, evidence of dynamic internal pressure, even at rest. This cannot be observed in a cadaver.

Tissue tension is a factor that must be taken into consideration. When the skin is incised during surgery, the dermis retracts more than the epidermis because it is under greater tension. It is also easy to observe that fatty lobules are being pushed towards the surface until they gradually emerge. Another example is the section of an aponeurosis using a scalpel, which causes the edges to retract like an elastic band, as seen in Figure 7.

Human beings therefore have an internal pressure, which means that, even at rest, living bodies exist in a state of dynamic equilibrium that cannot be observed in a cadaver. Living bodies are in a permanent state of tension, with tissues that are pre-stressed, and under the influence of gravity, the most significant of forces to which they are subjected.

**Figure 8:**
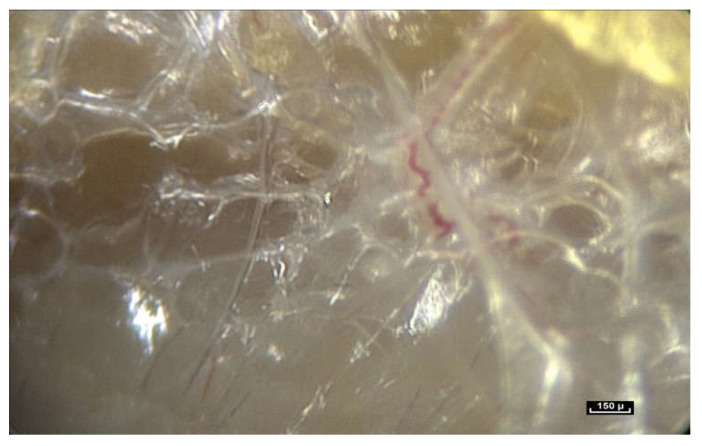
(Video S8) Sharp traction on this fibrillar network causes curious movements to occur due to the bursting of microvolumes at atmospheric pressure, in the operating theatre demonstrating the existence of hydraulic systems under different levels of pressure.

A permanent struggle of physical forces takes place within the living human body. For example, during endoscopy, sharp traction on this fibrillar network causes curious movements to occur due to the bursting of microvolumes at atmospheric pressure, demonstrating the existence of hydraulic systems at different levels of pressure (Figure 8).

### 3.4. Fascia/Connective Tissue Fibers Demonstrate Nonlinear Behavior

During a flexion–extension movement of a finger, for example, the tendon can be seen to move beneath the skin, but the tissues around the tendon do not move. The movement of the tendon and the constraint on the surrounding tissues are absorbed. One of the aims of this study is to attempt to understand the mechanism responsible for the gliding of structures such as tendons and muscles without any dynamic influence on the surrounding tissues.

**Figure 9:**
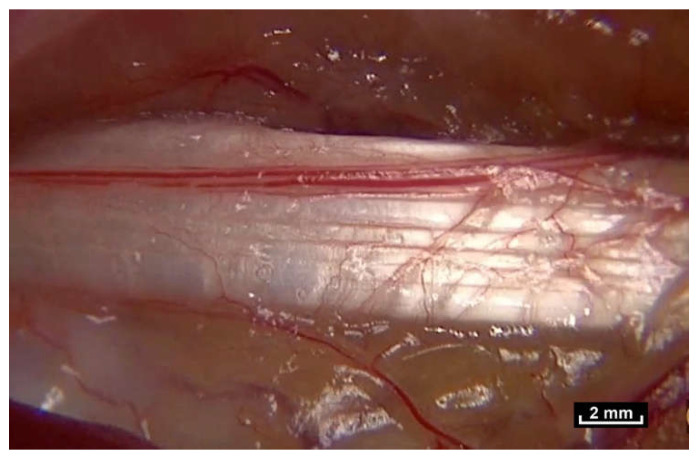
(Video S9) Some vessels do move with the tendon, but others are slower. There is no apparent synchronization or coherence and it can be seen that vessels move at different speeds, with a physical link between the vessels, but this is not a direct link. Once again, movement is irregular and nonlinear. There appears to be some sort of force absorption system with the surrounding tissues.

Furthermore, it would be logical to expect that during flexion–extension, the vessels would move at the same speed as the tendon, simultaneously, and in a linear progression. However, this is not what is observed. Some vessels do move with the tendon, but others are slower (Figure 9).

There is no apparent synchronization or coherence and observation reveals that vessels move at different speeds. There is a physical link between the vessels, but this is not a direct link. Once again, movement is irregular and nonlinear. This leads to the hypothesis that there is some sort of force absorption system. This is undoubtedly a disturbing observation, especially as this is taking place in a homogeneous tissue mass. Intra-tissue endoscopic observations do not concord with the received wisdom of separate, distinct tissue layers or strata.

### 3.5. Fascia/Connective Tissue Fibers Are Capable of Specific Movements That Ensure the Mobility of the Tissues—Fractalization

A constant observation is that fibers under stress will always unfold in the direction of the imposed constraint, in a non-linear pattern that seems anarchic, and beyond conventional understanding, but nevertheless allows mobility without rupture, and absorption of the force, while at the same time maintaining physical continuity and tissue memory. But the main question still remains: how does this fibrillar network that is mobile, display a specific type of biomechanical behavior, and how does this gliding system of the fascia/connective tissue function?

To explain how fascia/connective tissue glides, the academic community resorts to a number of mechanistic constructions inspired by industrial mechanics of the nineteenth century, using virtual spaces, pistons, separate layers, drawer-like arrangements and superimposed rings of decreasing diameter [26,27,28,29].

But this is not what is seen during intra-tissue endoscopy. On closer inspection with the endoscope, a network of fibers and small polyhedral forms can be seen, with surfaces that reflect the cold light emitted by the endoscope. These two elements, fibers and reflective surfaces, which were already seen in the pre-aponeurotic zone, seem to occupy all the available space revealing an irregular, seemingly haphazard organization, with very few cells.

**Figure 10:**
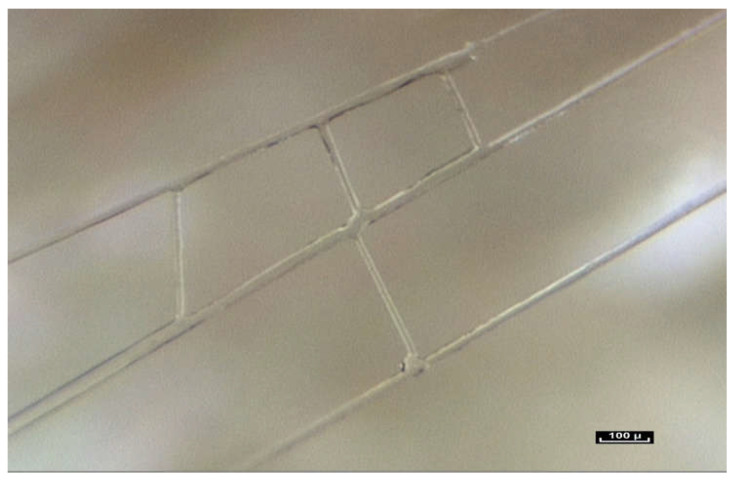
(Video S10) The fibers display three basic movements: Gliding, then dividing and then lengthening, all within a few tenths of a second. One fiber at first appearing to glide over the other fiber and then dividing into two, and finally another one lengthens. Animated diagram to illustrate this.

By meticulously screening a video sequence of endoscopic exploration of the zone between the Serratus Major and Latissimus Dorsi muscles, it was possible to observe a single fiber changing its appearance—at first dividing into two, and then appearing to glide over the other fiber. This was the first time fibers were seen to move in this way. The video sequences recorded during this study reveal that fibers display three basic movements: gliding, dividing and lengthening. The following video shows these three movements occurring within a few tenths of a second (Figure 10).

Lengthening is estimated to be between 10 and 30% of the original length of the fiber, and seems to be the most common movement. It takes place either within collagen fibers with a smooth surface, or within fibers that have rings that expand and retract, similar to the rings of an earthworm (a working hypothesis is that this is enabled by a higher concentration of elastin fibers; however, further research is necessary to verify this). One can also observe the gliding of one fiber along another fiber. For this movement to be carried out, it is suggested that the only plausible explanation would be instantaneous depolymerization and repolarization at the mobile junction of the two fibers. Close observation of this video sequence suggests that the movement is potentially partially reversible. Finally, the division of fibers can be observed in this video sequence. Fiber division appears to be unpredictable. Sometimes fibers can be seen to divide into two, three or four sub-fibers instantly, and then can come together and reconstitute a fiber similar to the source fiber. At other times, the end result of fiber division is the apparition of a completely different fiber.

It is suggested that this inherent capacity of fiber division enables the fibrillar network to deal instantly with an imposed force whether intrinsic or extrinsic. The force appears to be dispersed throughout the fibrillar network. In this way, the fibrillar network is able to respond to any constraint, either from the inside via the muscles or from the outside, in all three dimensions. This process is understood by the authors to be a process of fractalization.

Fractalization is a widespread phenomenon in anatomy. In this way, a large surface can be contained within a small volume, thus providing a larger surface area for exchange. This is the case with the alveoli in the lungs but also the intestinal villi. We find similar arrangements in tendons. It is important to note that the irregular fractalization within this fibrillar network is not inert; it is dynamic. We propose the term “dynamic fractalization” to describe this phenomenon.

Another important observation is that there are some fibers within this fibrillar network that appear to be stable, and do not move, or at least do not respond to an imposed force. They do not take part in the dispersion of the force, but appear to stabilize.

These observations suggest that fascia/connective tissue plays a role that goes far beyond that of a simple packaging tissue. The non-linear, apparently unpredictable behavior of fibers and fibrils within the fibrillar network is incompatible with traditional, accepted biomechanical musculo-skeletal models of human movement, and yet it clearly plays an essential role in movement [30].

### 3.6. Fascia/Connective Tissue Fibers Act as an Absorbing System That Ensures Flexibility

These observations suggest that the fibrillar network acts as a force absorption system in addition to providing a framework or scaffolding for cells. We advance the hypothesis that this network extends throughout the body, providing a framework or scaffolding for cells, regardless of their size, location or specific function.

**Figure 11:**
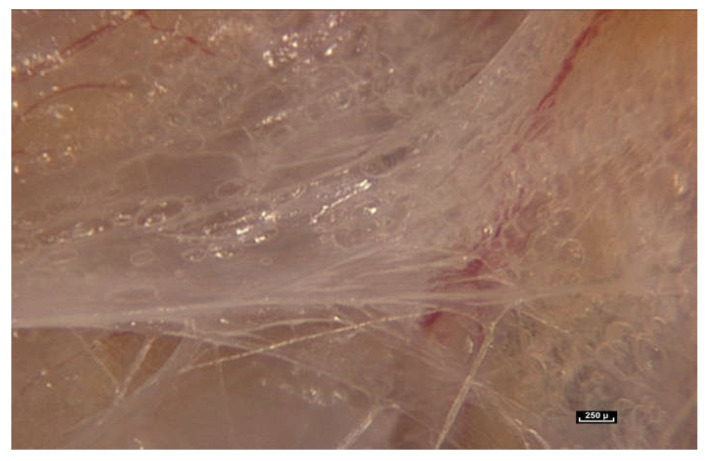
(Video S11) When tension is applied the fibers are stretched as the force increases. The adjacent linked element is subjected to tension. The fibers become more aligned in the direction of the applied force. The constraint is gradually diffused, dispersed and absorbed by the fibrillar network due to the capacity of the fibers to distend, divide and glide along each other.

Each fiber is thought to be prestressed and connected to its neighboring fiber. When tension is applied to the link, the adjacent element is subjected to tension. The fibers are stretched as the force increases. They are also seen to become more aligned in the direction of the applied force. The ability of certain fibers within the network to lengthen, divide and glide along each other appears to ensure that the force is gradually diffused, dispersed and absorbed by the fibrillar network (Figure 11) [31,32].

Close observation of films obtained by intra-tissue endoscopy suggests that the greater the distance from the force, the less impact it has on the fibers. The force is therefore absorbed, gradually decreasing in intensity, thus minimizing the impact on the surrounding tissues. It would appear that the fibrillar network behaves in this way in order to avoid rupture. This biomechanical method of force transmission, of force absorption, has never been described.

### 3.7. Fascia/Connective Tissue Fibers Are Intercrossed and Interwoven in Three Dimensions to Create Microvolumes—The Organization of Living Matter Can Therefore Be Interpreted in Terms of Volume, Form and Mobility

(i) An irregular polyhedral fascial fibrillar framework

As the magnification is increased, it can be seen that fibers are arranged in a dispersed, apparently haphazard and disorderly configuration in three dimensions. They appear to be everywhere, and there are no Euclidean shapes.

**Figure 12:**
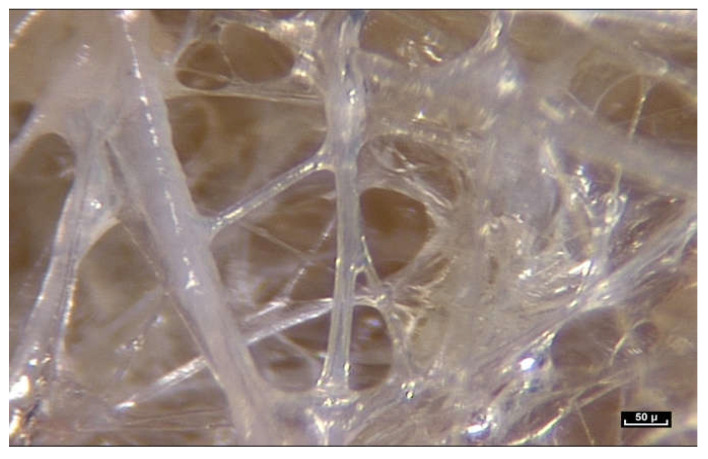
(Video S12) (×100 magnification) The fibers are arranged in a completely disorderly fashion. They may have multiform connections that are very dense and irregular. They divide into smaller-diameter fibrils of a few microns in diameter with extremely variable lengths of 20 to 100 microns and of irregular thickness, giving a disordered and chaotic appearance.

At first sight, all the fibers look alike. But on closer inspection, there is great diversity in the shape and form of the fibers. As the endoscope travels along these structures, constant observation is of the same fractal arrangement everywhere: large fibers intersected by smaller fibrils (Figure 12). The fibers and fibrils may have quite large diaphanous surfaces, reflecting sharp edges but they can also be narrow, long or short, swollen or cylindrical.

The fibers divide into smaller fibrils with a diameter of a few microns and extremely variable lengths of 20 to 100 microns. Their disposition is vertical, oblique, or transverse. They are either close together or far apart, and of varying density. The overall appearance is that of a disorderly, haphazard arrangement of the fibers.

(ii) Microvolumes

**Figure 13:**
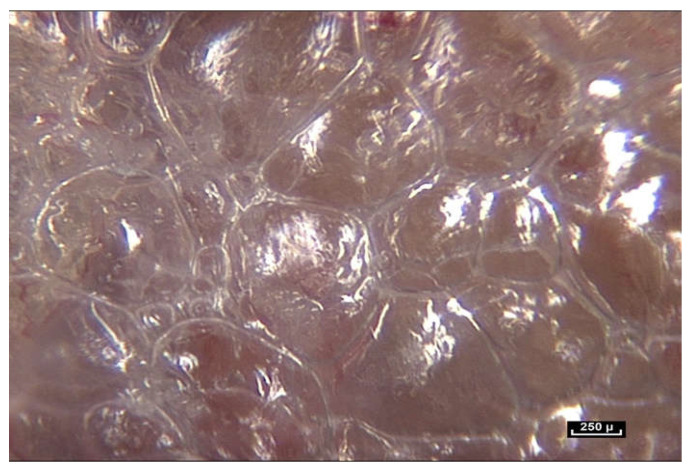
(Video S13) (×40 magnification) As we move the endoscope closer to these areas the light emitted by the endoscope is reflected from the glistening facets of the microvolumes, that resemble a pile of mirrors dumped arbitrarily in a heap. These forms that we see are polyhedral, irregular microvolumes but the physical status of a microvolume is itself unstable. Animated diagram to illustrate.

In our opinion, the notion of volume is crucial because it is closely linked to the notion of form. We have observed small polyhedral forms with surfaces that reflect the cold light emitted by the endoscope. This is a constant observation in the pre-“deep fascia” areas. Microvolumes are formed by the interweaving and intercrossing of fibers in three dimensions. They have irregular polyhedral shapes like everything seen so far—all different from one another. It is important to reiterate that any surgeon can see microvolumes during surgery. They are visible to the naked eye (Figure 13).

These irregular microvolumes were called microvacuoles at the beginning of our endoscopic exploration, because there was nothing visible inside them. They contain a hydrated proteoglycan gel (70%). Their lipid content (4%) is high and they are composed of collagen types 4 and 6 [16]. As a result of their strong negative charge, glycosaminoglycans (GAGs) attract counter-ions and water molecules into the tissue. These are fluids and gels—not simple water, and the arborimorphous structure of the GAG ensures a certain physical continuity [33,34].

By accumulation and superposition, these microvolumes with polyhedral patterns build elaborate forms that seem to vary greatly depending on their location. But these volumetric units can also be filled with cells that are generally in the same order of magnitude, that is to say between 20 and 100 microns. On the whole, there is a permanent adaptation of the volume according to peripheral pressures.

This complex microvolumetric architecture appears to play a role in fluid distribution in the body. The human body is composed of 75 to 80% of fluids. These fluids do not move around freely because when an incision is made in the skin of a living organism in the area of a bloodless field that has been created by the application of a tourniquet, there is no significant secretion of fluids. The small amount of fluid secreted can easily be wiped away with a compress. This is called Lymph but it is in fact the extracellular fluid. Liquids do not escape violently like the bursting of a balloon full of water. The liquids or fluids appear to be contained within this fibrillar network, with constant pressure and changing microvolumes [35,36,37].

These microvolumes are highly mobile and certainly not hermetically sealed, due to the tensioactive nature of the interfibrillar framework that is able to adapt to local variations in pressure and volume. In some circumstances this water component is excessive. The simple observation of tissue flooding in oedematous tissue is evidence of this. The drying out of the microvolumes after 1 h’s exposure to scialytic lighting during surgery shows that the omnipresence of fluids in all living tissues, and the predominantly liquid nature of living matter cannot be ignored.

(iii) Fibers within this apparently irregular fibrillar framework display surprisingly efficient mechanical behavior.

The result of the movement of these fibers in three dimensions, combined with changes in volume and pressure in the microvolumes created by the interweaving of fibers, is the ability to adapt to any type of external or internal stress without a break in structural continuity [34,38,39,40,41].

This type of mechanical functioning challenges the traditional mechanical models already described, and is evidence of a method specific to living matter that opens up new avenues for further research and investigation.

### 3.8. The Relationship Between This Network of Fibrils and the Cells

Observation of this fibrillar network reveals the intimate relationship between the fibrillar network and the cells. The fibrillar network extends into and structures the extracellular matrix (ECM) between cells at all scales.

Cells are embedded in this network, which appears to provide the three-dimensional architecture of the ECM, the natural environment of cells in the living body. This is evidence that a cell cannot exist outside this fibrillar network. In the following video, the fibers and fibrils of the fibrillar network penetrate a cellular cluster of fibrocytes and merge completely into it.

**Figure 14:**
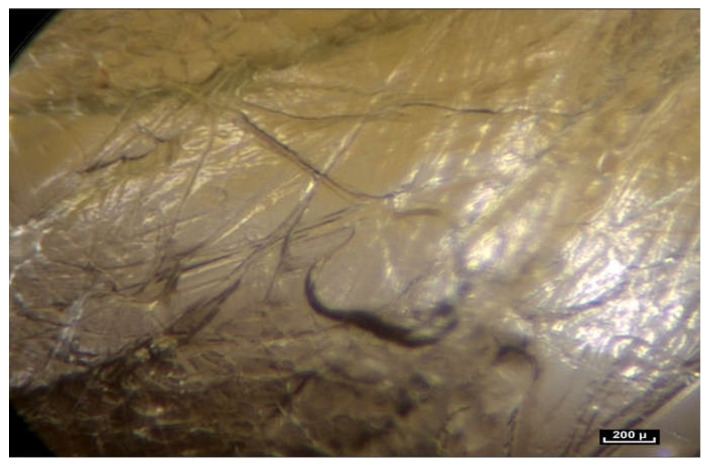
(Video S14) (×60 magnification). There is no doubt that the position of cells in space is determined by this interwoven latticework of intersecting fibrils. The cells seem to be nestled in this network and to be entirely part of it. This would appear to be evidence that a cell cannot exist outside this fibrillar network. Animated diagram to illustrate.

**Figure 15:**
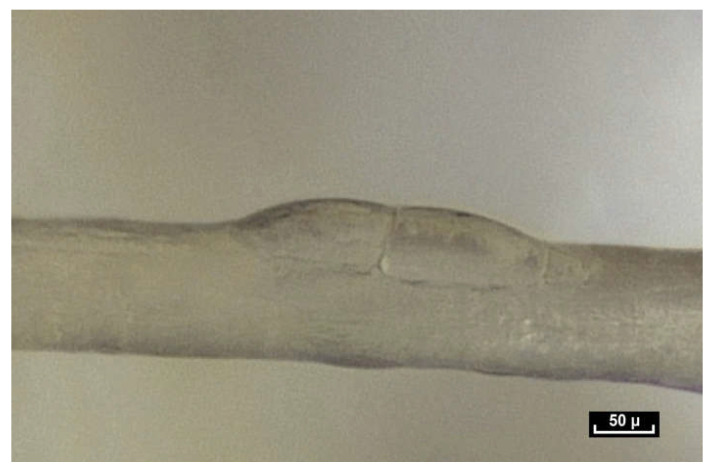
(Video S15) Along the fibers, cells are found either in pairs, like ladybirds on a blade of grass or in small groups.

There are no visible gaps or tissue barriers in the living tissue filmed during this study. Cells and connective tissue appear to be inseparable and the fibrillar network is seen to influence the shape and mobility of the cells [42,43,44,45,46] (Figure 14). It illustrates the investigative nature of this research. Without a pre-planned scenario, the operator could have chosen to go to the left but we went to the right. This particular fiber was chosen to film because the focus and contrast were good. Suddenly two ladybird-like shapes came into view on the fiber. There is no doubt that these are cells, and it was pure chance to have seen them. This unexpected discovery raised even more questions. What are these cells doing here? Why have they moved away from the main group of cells, and what is the role of the fiber? (Figure 15).

**Figure 16:**
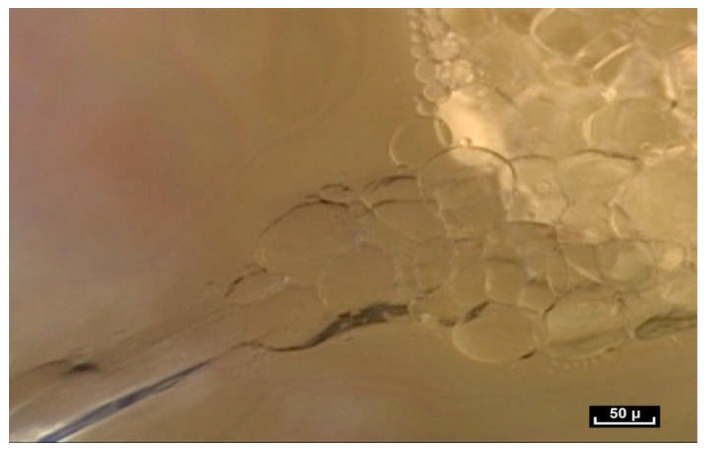
(Video S16) The fibers penetrate the cell groups and are therefore interconnected. The fibers seem to be swallowed up by the cell groups via the intercellular membranes, and this fusional relationship between the fiber and the cell is obvious.

Do cells use fibers to migrate? Do they move along the fibers of the fibrillar network? It was at first thought that these cells were gliding along the fibers like a snail on a leaf. However, it is clear that this cannot be the case. Fibers can be seen to penetrate these groups of cells and so cells and fibers are therefore interconnected. The fibers seem to be engulfed by the cell groups via the intercellular membranes. Once again, it is important to highlight this fusional relationship between the fiber and the cell (Figure 16).

It would appear that the fibrillar network, cells and vessels are indissociable and that cells are not the only anatomical structures responsible for the shape of a body.

### 3.9. Fascia/Connective Tissue Fibers Behave in Ways That Do Not Conform to Traditional Science

(i) The fascial fibrillar network displays structural irregularity.

Inside the body, there are not many horizontal, vertical or oblique structures with repeated angulations [47,48].

At the macroscopic level, Leonardo da Vinci’s Vitruvian Man represents the human body in terms of perfect symmetry, but in fact, this is not entirely correct because the heart is on the left, and the liver, with the gall bladder, on the right, etc. This supposed regularity, this apparent symmetry, is never perfect—only in appearance, and in reality, totally asymmetrical. At the microscopic level, fibrillar diversity, irregularity and lack of symmetry are a constant observation. Further research is needed to understand why the arrangement of fibers within this fibrillar architecture is irregular.

(ii) Fascia/connective tissue fiber behavior is uncertain, instantaneous and unpredictable.

**Figure 17:**
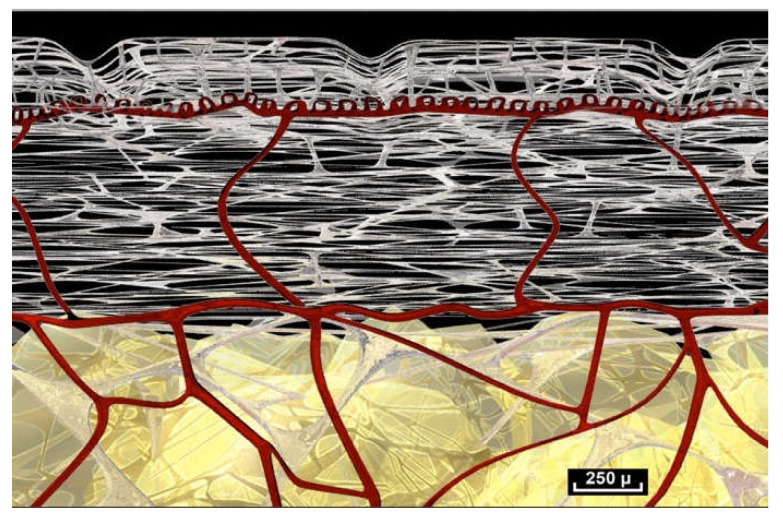
(Video S17) Global diagram of the fibrillar intra-tissue organization showing the fibrillar network at different levels.

The concept of layers, strata and separate planes would therefore be rendered obsolete by the information provided by intra-tissue endoscopy (Figure 17). And, if this is validated by further research, should be replaced by the observable reality of a single, common fibrillar network inhabited by cells with different densities that gather together to perform specific functions. Anatomy textbooks still describe highly stratified layers of tissue, as do recent fascia research papers [1,2,3].

**Figure 18:**
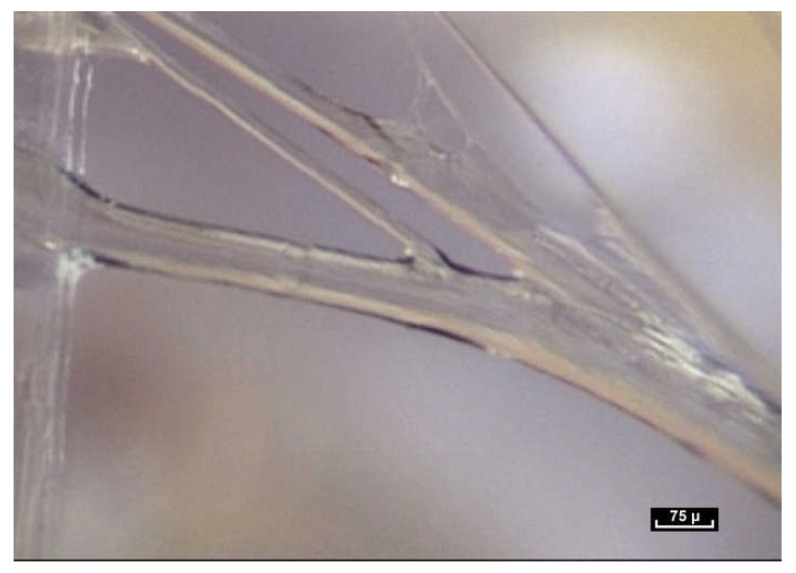
(Video S18) The migration of a fiber along other fibers. These junctions are mobile. One of the two fibers glides along the other one. In this way, energy is dispersed and absorbed throughout the fibrillar network. As soon as movement begins the fibrils respond by stretching out and lengthening. This ability to lengthen can be clearly seen.

Fractals and irregularity are constant observations. A further observation is that of unpredictability, as demonstrated in Figure 18.

A fiber within the fibrillar network can be seen moving along another, more vertical fiber. The fiber seems to be hesitating, ready to offer numerous biomechanical possibilities, moving up and moving down. It is not possible to anticipate its trajectory. None of the possible outcomes is predictable [49,50].

**Figure 19:**
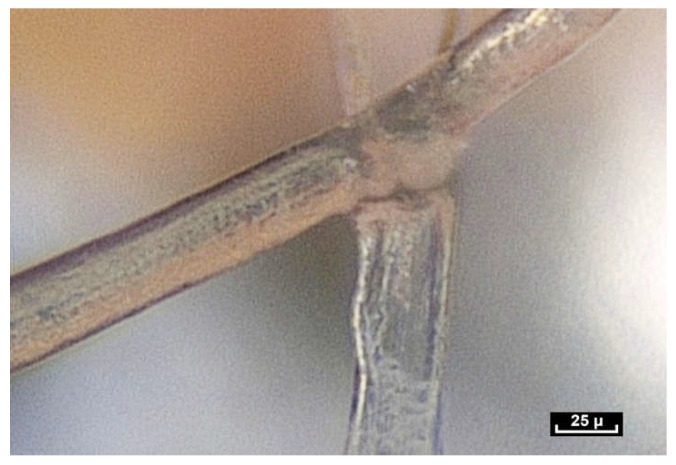
(Video S19) In this case, initially, there are 3 fibers, then 4, then 2, but it is impossible to predict or anticipate the movements. Here, one can see 2 large fibers. One of the two vertical fibers divides into three sub-fibrils, and you might think that one of the three fibers is going to take part in the action. But finally, it is a 4th fiber that is not initially visible, not predictable, that will interact.

In this video (Figure 19), there are initially three fibers, then four, then two, but it is impossible to predict or anticipate the movements of the fibers. Here, two large fibers are visible. One of the two vertical fibers divides into three fibers.

**Figure 20:**
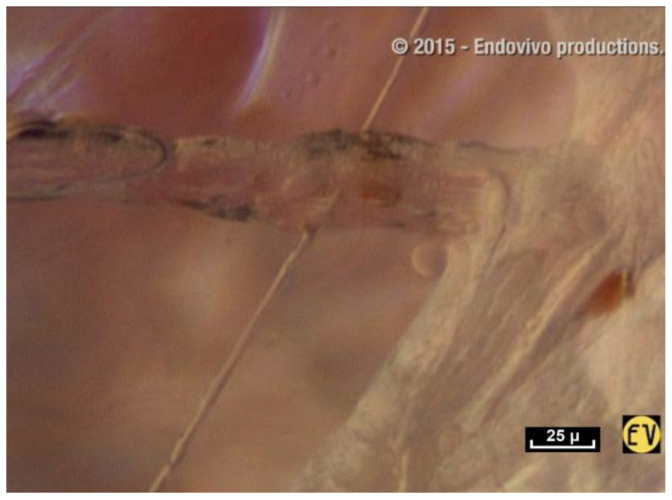
(Video S20) Sometimes, fibers are fixed and seem to stabilize the framework.

Fractalization is not always indeterminate. It can be limited to two planes, as in this fiber with what are thought to be elastin rings which can only extend or retract. However, some fibers appear to be fixed. In this video, two large fibers look as if they might separate or move along each other, but nothing happens (Figure 20).

**Figure 21:**
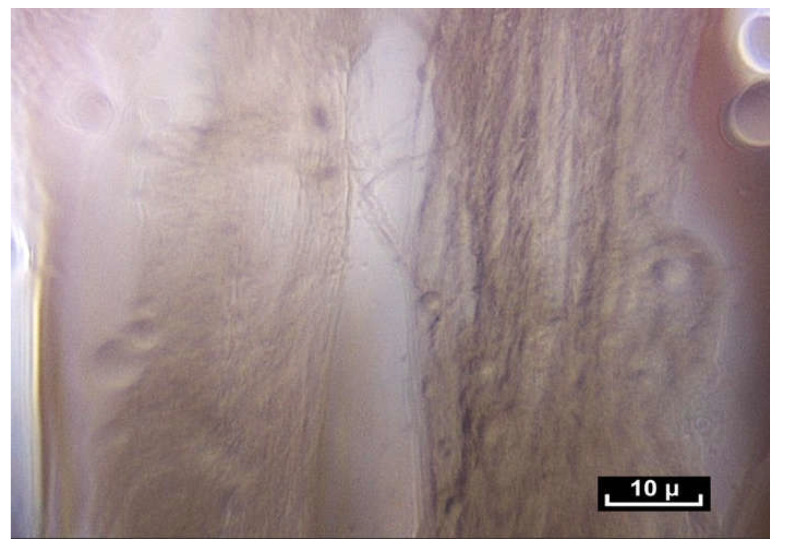
(Video S21) (×120 magnification) It would seem that these movements of division and gliding could occur in distinct ”separation zones”. This raises the question of morphological determinism, i.e., could these zones be predetermined or not?

**Figure 22:**
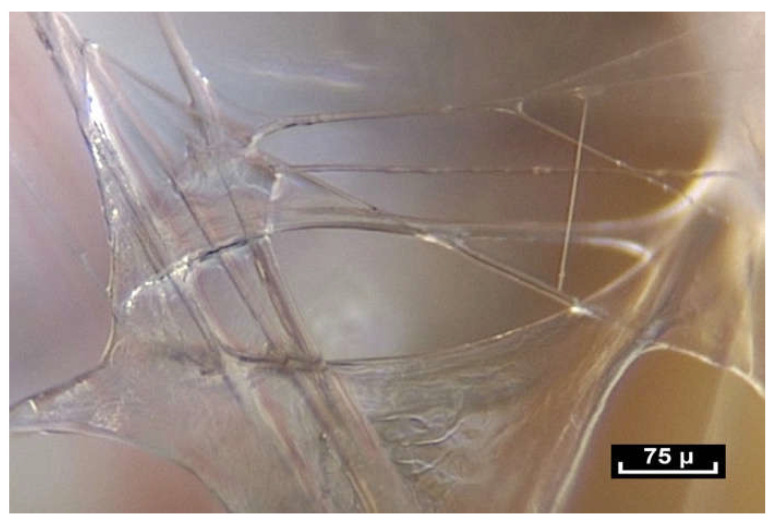
(Video S22) No movement of any fiber can be anticipated or predicted, as can be seen in this case. The dynamic potential of the combination of these three mechanical solutions is incalculable. The combined action of these three distinct, yet closely related, types of fibrillar behavior enables the fibrillar network to adapt to the constraint in all three dimensions, while at the same time dispersing and reducing the force of the constraint and preserving the capacity of the structures to return to their resting positions. Animated diagram to illustrate.

**Figure 23:**
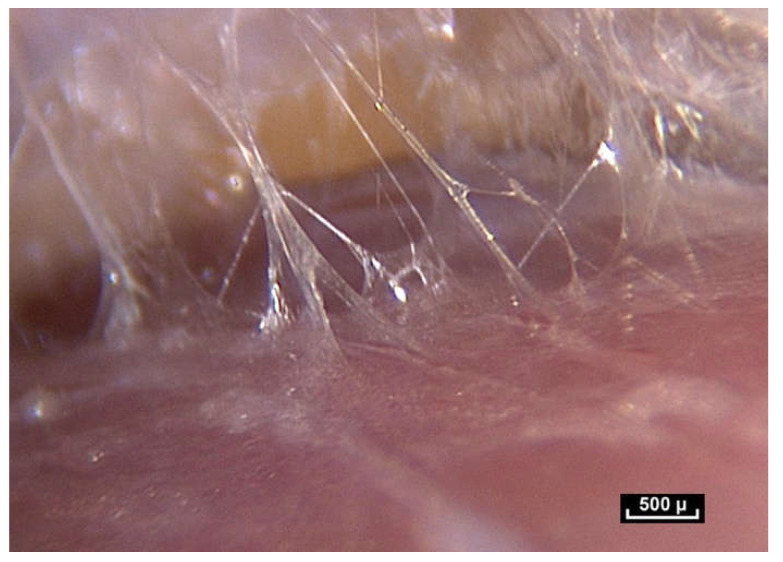
(Video S23) This set of trajectories and potentialities means that the mechanical solution is totally unpredictable and non-determined. The conclusion, illustrated by these images, is that a gesture cannot be repeated identically, in terms of its fibrillar execution. This is impossible because the interplay of the fibers will inevitably be different and so we can put forward the hypothesis that the gesture is unique in time but also in space.

The next question could be the following: are the splitting zones of the fibers programmed into the molecular architecture? In this case, for example, the division will take place in this area. But is the division predetermined by the alignment of the collagen molecules, or is it decided on the spur of the moment, and subject to the constraint at that particular moment? (Figure 21) No movement of any fiber can be anticipated or predicted can be seen in the following video sequences (Figure 22 and Figure 23).

(iii) Global coherence

Two conclusions can be drawn from these observations. The first is the expression of architectural coherence thanks to the association of a body-wide fibrillar network and the formation of microvolumes [49].

The same fibrillar system was encountered in every area investigated by intra-tissue endoscopy during this research. In light of these observations, it is proposed that the fibrillar system is present throughout the body, whatever the tissue type and location. If this is the case, it would be possible to describe the overall constitutional architecture of the living body in terms of its fibrillar framework. We propose that this same architecture would appear to play an essential role in movement and mobility. It would also appear to provide a framework for cells. The fibrillar network is of uniform construction, an interweaving of fibers and fibrils in three dimensions.

There is no observable variance from the basic architecture, simply local adaptations according to role and topography. Classifications, such as for example, of different types of fascia seem inadequate when confronted with the global, ubiquitous architectural characteristics of the fibrillar network.

**Figure 24:**
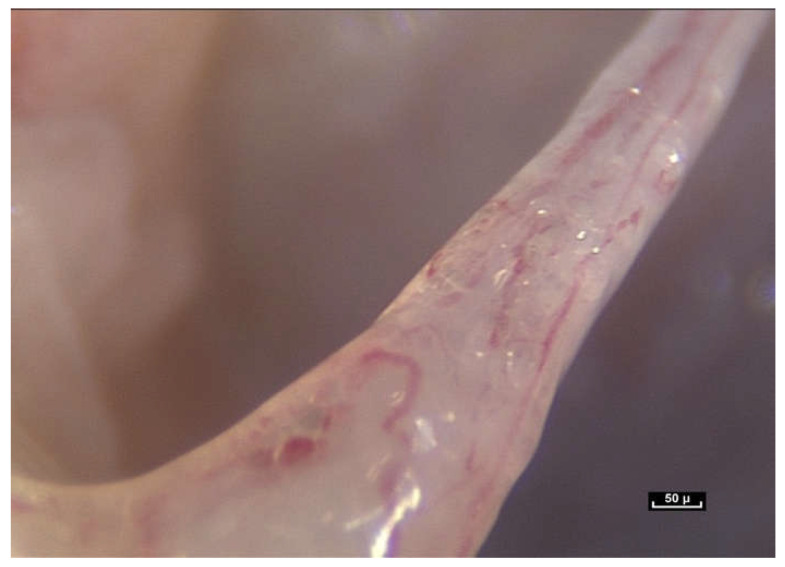
(Video S24) This same architecture is used either for mobility or as a cellular habitat. Sometimes you can even see the two combined, as in this case, where a fiber provides shape, mobility, flow of nutrients and cell housing.

The mobile component in the gliding (sometimes referred to as sliding) areas appears to be essentially made up of fibers and glycosaminoglycans, with only a few cells. This fibrillar network appears to play a role in absorbing forces and spreading the load of an imposed constraint, while at the same time providing a supporting framework for cells, as in this sequence where a fiber provides shape, mobility, flow of nutrients and cell housing (Figure 24).

## 4. Discussion

The human “fascial system” is still defined as a “layered body-wide multiscale network of connective tissue” [10]. However, intra-tissue endoscopy suggests that, through the interconnectivity of these tissues, there are no separate, distinct layers of fascia in the living body, as in a thousand-leaf cake. “Layers” of fascia can be artificially created by dissection, or observed in a cadaver without tissue tension, but this implies that the fibrillar network that creates the interconnectivity between these so-called “layers”, which are themselves part of the global and architectural fascial system, has not been taken into consideration. This fundamental difference in the conception of the organization of living matter is essential, and must be taken into account in any attempt to reach a consensus on the definition of the” fascial system”.

### 4.1. The Behavior and Architecture of the Fibrillar Network Opens a New Way of Thinking About the Organization of Living Matter

Intra-tissue endoscopy has revealed an internal architecture of fibers and fibrils that move, form microvolumes, are always in continuity, and are arranged in a disorderly, dispersed configuration. They appear to always unfold and align themselves in the direction of an imposed force constraint, in a non-linear way but nevertheless enable mobility without rupture while ensuring absorption of the force. At all times, the physical continuity of the fibrillar network is maintained. This seems to be beyond our conventional understanding of biomechanical models of movement [51,52,53,54,55]. There appears to be an underlying order within the apparent heterogenic, disorderly arrangement of fibers.

These films obtained by intra-tissue endoscopy reveal that fibrillar architectures from the surface of the skin to deep inside the bone can be seen to adopt the same construction system with local functional differentiations. These observations point towards the hypothesis that a living body is sustained by an architecture, a fibrillar network or a framework. The accepted role and behavior of connective tissue would then appear to be insufficient (Video S17).

### 4.2. Fascia/Connective Tissue Fibers Are the Structural Architecture of the Body, the Connective Tissue Is the Constitutive Tissue

Based on these observations, it is suggested that this fibrillar network is an essential component of the body’s architecture, because it can contain cells, as well as the elements necessary to ensure mobility, adaptation and force absorption. We suggest that it is responsible for the maintenance of form. The movement and adaptability of the fibers can be seen to be rapid, fluid and harmonious. We propose that the gliding movements of anatomical structures relative to each other are not carried out by separate layers of tissue, but by this mechanism of fibers which are able to divide and glide along each other. This enables them to adapt in an instant to any force or constraint in all three dimensions without rupture. In light of these observations, the organization of living matter in separate tissue layers would appear to be an obsolete concept. This would represent a significant paradigm shift.

### 4.3. The Emergence of a Post-Vesalian Anatomy

The body cannot be considered as a machine made up of separate parts, or like a cake with icing between separate layers. The concept of layers, strata and separate planes must be considered to be obsolete, and needs to be replaced by the observable reality of a single, common fibrillar network. Our anatomy textbooks still describe highly stratified layers of tissue. The anatomy described by Vesalius and our elders must now be reconsidered in light of current scientific thinking that integrates life sciences with modern physics and new mathematics [56].

### 4.4. Paradigm Shift

The term ”fascia” is currently reserved for the dense fibrous structures in the body, such as fascia superficialis, fascia profunda, fascia Lata, the plantar aponeurosis, the lumbosacral fascia, etc. This definition is inevitably underpinned by the anatomical location in the body, and is limited to macroscopic structures that are perfectly visible to the naked eye [57,58,59,60].

This definition was subsequently extended to include loose areolar connective tissue. However, intra-tissue endoscopy has now provided evidence that all these structures are made up almost exclusively of fibers of varying density. They are all examples of multifibrillar structures and appear to be an integral part of a vast body-wide fibrillar network.

We believe that this has been demonstrated by intra-tissue endoscopic explorations that are scientific, precise and easy to reproduce.

This hypothesis is in accordance with the conclusions of a recent paper that proposes “an updated fascia-centric interpretation of architectural anatomy” and describes the fascial system as a “tensioned fibrous network encompassing a complex heterarchy of regionally specialized compartments” each with its own parenchyma driven characteristics “that contribute to the functional whole” [55].

Mechanisms of dissipation and absorption of an applied force by the fibrillar network to avoid rupture have been described in this paper, based on observation of video sequences obtained by intra-tissue endoscopy.

Similar conclusions were reached by the authors of a recent paper that describes “a mechanism, not yet fully understood”, of the dissipation of forces, of either internal or external origin, that are “constantly generated and transmitted throughout tissue”. The paper states that reducing and maintaining internally or externally originating forces to a level that does not damage the structures involved “is achieved by dissipation, transduction and transferring the force in multiple dimensions”. This must occur “in a continuous and consistent way from the cellular level to the entire body”. The purpose of this mechanism is to “prevent structural damage to cells and tissues”.

## 5. Conclusions

Intra-tissue endoscopy has revealed that a fibrillar network can be found in all tissue types: muscles, periosteum, bone and the fatty lobules in the hypodermis. This research has also highlighted the intimate relationship between the fibrillar network and cells. These observations would suggest the existence of a continuous, global, body-wide fibrillar network.

The same fibrillar organization was observed in all the anatomical areas that were explored, without exception: hand and leg tendons, the scalp, the foot during toe transfers, the abdomen, in the vicinity of the lumbar spine, but also between the breast and the rib cage and within all the tissues explored from the surface of the skin to the periosteum in a completely ubiquitous manner. This was not the case regarding cells.

In light of these observations, it is proposed that the organization of living matter could be defined as a fibrillar whole, a global framework starting at the surface of the skin and continuing through the dermis, hypodermis, muscles, tendons, vessels, periosteum and bone, in which the various cell types are housed and are able to carry out their specific functions within this global architecture. The received wisdom of separate organs joined and held together by inert connective tissue would render traditional models obsolete.

Anatomy could therefore be seen in a different light. This study provides evidence of structural coherence due to physical continuity, from skin to fat, tendons, muscles, nerves and vessels. The Vesalian vision of separate organs and the traditional compartmentalized division of the body into different layers, like a cake with layers separated by icing, would be rendered obsolete [10].

We, therefore, propose the hypothesis that this fibrillar network serves both generalized, global functions and specific, specialized local functions that have been dismissed and overlooked in the past. We believe that the relevance of this research crosses a wide range of scientific, medical and therapeutic disciplines. Hopefully, it will encourage wider inter-disciplinary dialogue and research in the future, improve communication between disciplines that tend to work in isolation and help to shape the discourse within the field of fascia research. The anatomy of the living human body can therefore be described differently [49].

This underscores the necessity for fascia research to evolve, expanding on the methods used in this research project and integrating modern in vivo imaging techniques [60]. The implications are that it is necessary to move beyond the confines of traditional methods of research such as dissection, ultrasound imaging, and the harvesting of tissue samples to be studied in laboratories, and to develop methods of real-time, in vivo study of fascia in its natural environment—in situ—inside the living body.

This research project must therefore be expanded and enriched by research from other disciplines. Only then could the importance of this ubiquitous irregular fibrillar network that has been neglected for such a long time be fully understood and recognized, because we believe that its role is at least as important as that of the cell.

## Data Availability

All videos and photos are currently stored in a database (https://we.tl/t-y23E78oSMl, https://we.tl/t-1PyXRsdwth). Accessed on 1 January 2025.

## Supplementary Materials

The following supporting information can be downloaded at: https://www.mdpi.com/article/10.3390/life15050791/s1.
